# Fufang Fanshiliu Decoction Revealed the Antidiabetic Effect through Modulating Inflammatory Response and Gut Microbiota Composition

**DOI:** 10.1155/2022/3255401

**Published:** 2022-10-10

**Authors:** Leyu Li, Guoxin Huang, Tingbo Chen, Hui Lin, Ruiyan Xu, Jinyan Cheng, Ying Hu, Weibo Dai, Gengting Dong

**Affiliations:** ^1^Pharmacology Laboratory, Zhongshan Hospital of Traditional Chinese Medicine, Zhongshan, Guangdong, China; ^2^Clinical Research Center, Shantou Central Hospital, Shantou, Guangdong, China; ^3^College of Health Industry, Zhongshan Torch Polytechnic, Zhongshan, Guangdong, China

## Abstract

**Background:**

Diabetes mellitus brings serious threats and financial burdens to human beings worldwide. Fufang Fanshiliu decoction (FFSLD), a traditional Chinese medicine formula showing great antidiabetic effects, has been used in clinics for many years.

**Objective:**

This study aims to explore the underlying therapeutic mechanisms of FFSLD in Type II diabetes mellitus (T2DM).

**Methods:**

Sprague–Dawley rats induced by high-fat diet feeding combined with streptozotocin injection were used to establish the T2DM model. All rats were randomly divided into 6 groups: control, model, metformin, high dosage, middle dosage, and low dosage of FFSLD. After 4 weeks of treatment, serum, intestinal mucosa, and fecal samples were collected for further analysis. ELISA was used to detect the diabetic-related serum indicators and proinflammation cytokines. Gene or protein expressions of mitogen-activated protein kinase (MAPK), interleukin 1 beta (IL-1*β*), transforming growth factor-beta (TGF-*β*), and tumor necrosis factor-alpha (TNF-*α*) in intestinal mucosa were analyzed by quantitative real-time polymerase chain reaction (RT-PCR) or western blot. 16s rRNA gene sequencing was used to detect the changes of gut microbiome in these groups. Intestinal gut microbiota (GM) composition was further analyzed according to the sequencing libraries.

**Results:**

FFSLD effectively recovered the diabetic-related biochemical indexes by reducing fasting blood glucose (FBG), total cholesterol (TC), triglyceride (TG), low-density lipoprotein cholesterol (LDL-C), insulin, and increasing high-density lipoprotein cholesterol (HDL-C). Furthermore, FFSLD significantly ameliorated the abnormal levels of proinflammation cytokines including IL-1*β*, IL-6, TNF-*α*, and TGF-*β*. In addition, the GM compositions of rats in control, model, and FFSLD treated groups were different. FFSLD significantly increased the relative abundance of *Lactobacillus, Akkermansia*, and *Proteus,* and reduced the relative abundance of *Alistipes*, *Desulfovibrio*, and *Helicobacter*. Moreover, these changed bacteria were closely related to the diabetic-related serum indicators and proinflammatory cytokines.

**Conclusion:**

These results suggest that FFSLD alleviates diabetic symptoms in T2DM rats through regulating GM composition and inhibiting inflammatory response, which clarify the therapeutic mechanism of FFSLD on T2DM and provide a theoretical basis for its further clinical application.

## 1. Introduction

Diabetes mellitus, a complex metabolic disease, has become one of the top 10 causes of death which makes a great threat and heavy financial burden to human beings worldwide [1]. International Diabetes Federation Diabetes Atlas reported that 4.2 million adults from 20 to 79 age died of diabetes in 2019 globally, equal to eight deaths per minute [2]. Long-term and uncontrolled hyperglycemia always causes a series of complications, for instance, diabetic nephropathy, cardiomyopathy, retinopathy, and neuropathy, which seriously affect the life quality and expectancy of diabetic patients [3, 4]. Therefore, it is urgent to investigate the pathological mechanisms and find out an effective therapy to control the development of diabetes as early as possible.

Recent studies showed that gut microbiota (GM) plays an important role in the progression of many metabolic diseases, especially diabetes [5, 6]. GM including probiotics, commensal bacteria, and potential pathogens coexist in the host gastrointestinal tract and interplay with the host metabolism and immune system to maintain host health. However, factors such as unhealthy lifestyles, antibiotic abuse, mental pressure, and food consumption could change the balance of the GM composition and lead to the occurrence and development of diseases. Dysbiotic GM aggravated the development of T2DM clinical symptoms via many mechanisms such as activating inflammatory response and altering mucosa permeability [7]. Reports showed that the genera, e.g., *Ruminococcus*, *Fusobacterium*, and *Blautia* were positively related to the progression of diabetes, however, the genera of *Bifidobacterium*, *Bacteroides*, *Faecalibacterium*, *Akkermansia*, and *Roseburia* were negatively associated with diabetes [8]. Metformin, one of the first-line drugs to treat diabetes, was proved to significantly decrease the relative abundance of *Intestinibacter* and increase the relative abundance of *Escherichia* spp. [9]. Other reports also showed that metformin possessed a similar effect of inducing the mucin secretion by *Akkermansia muciniphila* and could stimulate the growth of *A. muciniphila* [10, 11]. These results implied the strategy of diabetes therapy through the regulation of GM composition.

Fufang Fanshiliu decoction (FFSLD) is a traditional Chinese medicine formula composed of 10 different herbal medicines and has been used for diabetes therapy for more than seven years in clinics. Associated research studies showed that the drugs in FFSLD, for instance, *Psidium guajava* L. (Fanshiliuye) [12], *Morus alba* L. (Sangye) [13], *Euonymus alatus* (Thunb.) *Sied.* (Guijianyu) [14], *Coptis chinensis* (Huanglian) [15], *Crataegus pinnatifida B*. (Shanzha) [16], and *Monascus purpureus W*. (Hongqu) [17] displayed the direct or indirect effects of antidiabetes in preclinical and clinical studies; whereas the potential mechanism of FFSLD for diabetes therapy still needs further study. Recently, accumulating evidence showed that Chinese medicines, including the compounds, the extracts, and the formulas, can significantly regulate the GM composition to intervene the disease development [18–20]. Based on these research studies, we hypothesize that FFSLD revealed the antidiabetic effect through modulating GM composition. Here, in order to validate the potential mechanism of FFSLD revealed the antidiabetic effect, Sprague–Dawley rats fed with a high-fat diet simultaneously injected with streptozotocin (STZ) to establish the diabetic model and treated with different dosages of FFSLD for 4 consecutive weeks. At the end of the experiment, diabetic-related serum indicators including fasting blood glucose (FBG), insulin, triglyceride (TG), total cholesterol (TC), high-density lipotoprotein cholesterol (HDL-C), and low-density lipoptorotein cholesterol (LDL-C) were used to evaluate the antidiabetic effect of FFSLD. Moreover, feces were collected to investigate the change of GM composition by treatment of FFSLD using 16S rRNA sequencing.

## 2. Materials and Methods

### 2.1. Preparation of FFSLD

*Psidium guajava* L. (Fanshiliuye, #C22103062, 30 g), *Ficus simplicissima* Lour. (Wuzhimaotao, #C22108042, 20 g), *Morus alba* L. (Sangye, #C22110151, 15 g), *Codonopsis pilosula* N. (Dangshen, #C12108207, 30 g), *Coptis chinensis* F. (Huanglian, #C22105098, 10 g), *Atractylodes lancea* DC. (Cangzhu, #C22105060, 15 g), and *Crataegus pinnatifida* B. (Shanzha, #C22106013, 15 g) were purchased from Sinopharm Feng Liaoxing (Foshan) Herbal Pieces Co., Ltd (Guangdong, China), *Euonymus alatus* (Thunb.) Sied. (Guijianyu, #200601, 15 g) and *Monascus purpureus* W. (Hognqu, #210901, 15 g) were purchased from Zhixin Chinese pharmaceutical Co., Ltd (Guangdong, China), and *Zingiber officinale* R. (Ganjiang, #210801, 10 g) was purchased from Bencaotang Chinese pharmaceutical Co., Ltd (Guangxi, China). FFSLD was prepared by Zhongshan Hospital of Traditional Chinese Medicine (Guangdong, China) according to the standard production process. These herbs were immersed in water overnight and decocted two times (1st for 2 h and 2nd for 1 h). The decoctions were combined and freeze-dried to obtain the powder. Chromatographic peaks recorded by UHPLC-DAD assay (Supplementary file available (Here)) could be used for the quality control of FFSLD. The identification of 457 compounds was further performed by QTOF-MS as previously described [21]. In the animal study, the powder was reconstituted with physiological saline at the indicated concentrations.

### 2.2. Animal Experiment

Sprague–Dawley rats (160–180 g, half male and half female) were purchased from Guangdong Animal Center (batch number: SCKX2016-0041). All rats were housed in the IVC equipment and maintained at the constant temperature (22 ± 2°C) and constant humidity (55 ± 5%) with a 12-h light/dark cycle. All rats had free access to food and water. Prior to the experiment, rats were adaptively fed for 1 week. This study was approved by the Animal Ethics and Welfare Committee of Zhongshan Hospital of Traditional Chinese Medicine (AEWC-2016054, Guangdong, China).

**Figure 1:**
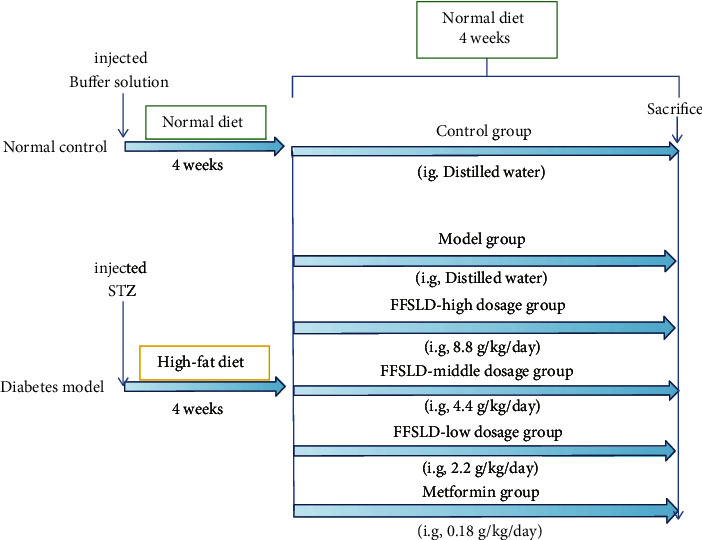
The schematic diagram of the animal study design. HFD/STZ-induced rats were used to evaluate the antidiabetic effect of FFSLD.

Animals were randomly assigned to diabetes (*n* = 30) and control group (*n* = 6). In order to establish a diabetic model, rats were intraperitoneally injected with STZ (55 mg/kg, single dose) in sodium citrate buffer after fasting for 12 h, and constantly fed with a high-fat diet (HFD; 15.5% protein, 38.8% fat, and 45.7% carbohydrate). The control group was injected with sodium citrate buffer and fed with normal chow (18% protein, 4% fat, and 5% crude fiber). 4 weeks later, the FBG of the rats more than 15 mmol/L were assigned to the diabetic group [22]. Then, these rats were randomly assigned to 5 groups: model (*n* = 6), metformin (0.18 g/kg/day, the equivalent dose used in the clinic, *n* = 6) [23], high dose of FFSLD (8.8 g/kg/day, *n* = 6), middle dose of FFSDL (4.4 g/kg/day, *n* = 6), and low dose of FFSDL (2.2 g/kg/day, calculated according to the extraction yield of crude material, the equivalent dose used in the clinic, *n* = 6). The rats have been orally treated with FFSLD or metformin for consecutive 4 weeks and were fed with normal chow during the experiment. The body weight and food consumption were monitored during the whole experiment. All rats were fasted overnight and anesthetized with sodium pentobarbital at the end of the experiment. Blood was collected from the tail vein, and serum was collected by centrifugation at 3000 r/min for 15 min at 4°C and then stored at −80°C for later studies. The schematic experimental plan of this study is shown in Figure 1.

### 2.3. Enzyme-Linked Immunosorbent Assay (ELISA)

FBG was measured with a glucometer (Shanghai Yuyue 301). The serum levels of TC, TG, HDL-C, LDL-C, insulin, IL-1*β*, IL-6, and TNF-*α* were detected by ELISA kits (purchased from Bioss Biotechnology Co., Ltd.) according to the manufacturer's instructions.

### 2.4. Quantitative Real-Time Polymerase Chain Reaction (RT-PCR)

RNA was extracted from intestinal mucosa using TRIzol reagent (Invitrogen, USA). Total RNA was reverse-transcribed to cDNA. RT-PCR was then used to determine the expression of target gene MAPK, with GAPDH as reference. Primer sequences: MAPK (F: *TTGCTCGGATTCCGCCATGA*, R: *GCTGGCTCTTTAGCAGCTTG*, 136 bp); GAPDH (F: *AGGGTCATCATCTCCGCCCC*, R: *TCCACGATGCCAAAGTTGTC*, 164 bp).

### 2.5. Western Blot Analysis

Rats' intestinal mucosa was homogenized on ice-cold water with lysis buffer (Beyotime, P0013 B). Equal protein concentrations were separated by SDS-PAGE (Beyotime, P0050) and transferred to the PVDF membrane (Beyotime, FFP24). After blocking 30 min with the QuickBlock™ Blocking Buffer (Beyotime, P0252), membranes were incubated overnight at 4°C with primary antibodies against MAPK (Abcam, ab185145), IL-1*β* (Abcam, ab200478), IL-6 (Abcam, ab259341), TGF-*β* (Cusabio, CSB-PA004279), and TNF-*α* (Abcam, ab66579). GAPDH (Abcam, ab181602) was used as a normalizing protein. Membranes were then washed 3 times with Western Wash Buffer (Beyotime, P0023C3) 3 times, followed by incubation with horseradish peroxidase (HRP)-conjugated secondary antibody for 2 h. Membranes were then washed 3 times with Western Wash Buffer (Beyotime, P0023C3), then incubated with luminescent substrate solution (Beyotime, P0018M). Protein bands were visualized by Chemiluminescence Imaging System (Bio-Rad, CA, USA) and analyzed with Image J software.

### 2.6. 16sRNA Sequencing

DNA was extracted from collected fecal samples using the EZNA tool DNA extraction kit (Omega, D4015), and the V3-V4 variable region was amplified with 16s rRNA primers. According to Gene Tools Analysis Software mixed PCR products inequal ratios, purification of PCR products, and sequencing libraries were generated using NEBNEXT Ultra DNA. Library quality was assessed using the Qu bit 2.0 Fluorometer and the Agilent Bioanalyzer 2100 system, then the library was sequenced on an Illumina Hiseq platform, 280 bp paired-end reads were generated following the sequencing libraries to analyze intestinal gut microbiota composition.

### 2.7. Statistical Analysis

Data were analyzed using SPSS (IBM, Armonk, USA) and GraphPad Prism 8.0 (GraphPad Software, San Diego, CA) and were expressed as mean ± SD unless otherwise specified. All endpoints are representative of at least 3 independent experiments and analyzed using one-way ANOVA with the post hoc Bonferroni test, with *p* < 0.05 as the cut-off for statistical significance.

## 3. Results

### 3.1. FFSLD Regulated Body Weight, Blood Lipid, and Blood Glucose in Diabetic Rat

**Figure 2:**
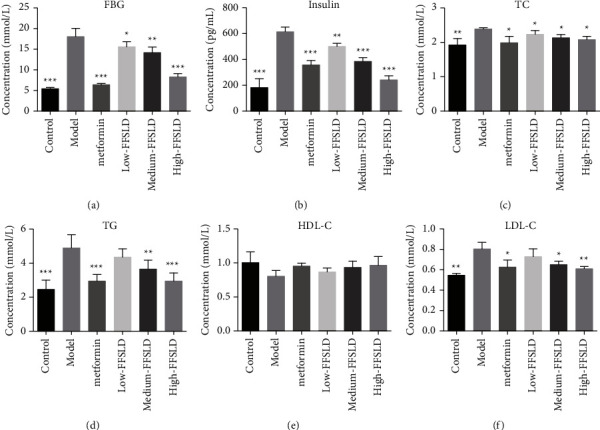
FFSLD ameliorated diabetic-related serum indicators of HFD/STZ-induced rats. (a) Fast blood glucose (FBG). (b) Insulin. (c) Total cholesterol (TC). (d) Triglyceride (TG). (e) High-density lipoprotein cholesterol (HDL-C). (f) Low-density lipoprotein cholesterol (LDL-C). Data are expressed as mean ± SD. ^*∗*^*p* < 0.05, ^*∗∗*^*p* < 0.01, and ^*∗∗∗*^*p* < 0.001, compared with the model group.

To evaluate the antidiabetic effect of FFSLD in the HFD/STZ induced rats, levels of FBG, insulin, TC, TG, HDL-C, and LDL-C were assessed. As shown in Figure 2(a), the mean FBG of rats in the model group was 18.22 ± 1.87 mmol/L, which was significantly increased comparing with that in the control group (5.51 ± 0.31 mmol/L) on the last day of the animal study. Compared with the model group, FBG of rats in the metformin group was 6.40 ± 0.30 mmol/L. FBG of rats in the FFSLD groups was significantly decreased in dose-dependent manner after 4 weeks of treatment. All these suggested both metformin and FFSLD significantly reversed the HFD/STZ increased FBG. Furthermore, the high levels of insulin in diabetic rats were significantly decreased after FFSLD and metformin treatments (Figure 2(b)). These results indicated FFSLD significantly improves insulin sensitivity in HFD/STZ induced rats. Besides, FFSLD remarkably alleviated HFD/STZ induced lipid metabolism disorders via decreasing TC, TG, LDL-C, and elevating HDL-C in a dose-dependent manner (Figures 2(c)–2(f)). These parameters were also significantly recovered by metformin. Altogether, these data validated the antidiabetic effect of FFSLD on regulation of glucose and lipid metabolism disorders in HFD/STZ-induced rats.

### 3.2. FFSLD Alleviated Inflammatory Response in Diabetic Rats

**Figure 3:**
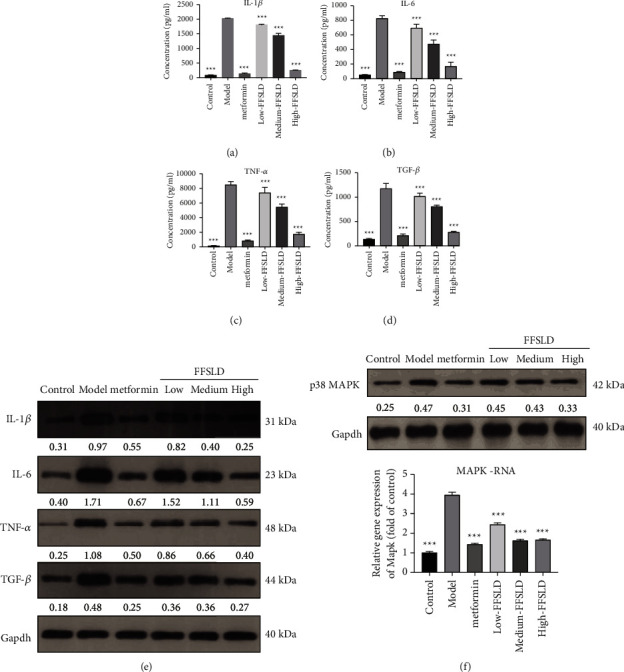
FFSLD alleviated inflammatory response of T2DM rats. ELISA for the serum levels of TNF-*α* (a), IL-1*β* (b), IL-6 (c), and TGF-*β* (d), western blotting for the expression of IL-1*β*, IL-6, TNF-*α*, and TGF-*β* (e), western blotting and RT-PCR for the expression of p38 MARK (f), data are expressed as mean ± SD. ^*∗∗∗*^*p* < 0.001, compared with the model group.

Inflammatory factors in the intestinal tract are stimulated by GM imbalance and are thought to influence the integrity of the intestinal barrier, which secondarily can influence nutrient absorption and metabolism. Firstly, the levels of TNF-*α*, IL-1*β*, IL-6, and TGF-*β* in the serum were analyzed by ELISA, FFSLD significantly decreased these proinflammatory factors in dose-dependent manner. Secondly, the protein expressions of TNF-*α*, IL-1*β*, IL-6, and TGF-*β* in the intestinal tract of diabetic rats were detected by western blot and the results showed that metformin and high-doses of FFSLD treatment blunted the expression of proinflammatory factors to the levels similar to the control group (Figures 3(a)–3(e)). In addition, p38 MAPK plays an important role in inflammatory response. Metformin and FFSLD treatment downregulated p38 MAPK expression in the intestinal tract which increased in the model group (Figure 3(f)), while the medium- and high-doses of FFSLD showed similar effects.

### 3.3. FFSLD Changed the GM Composition

**Figure 4:**
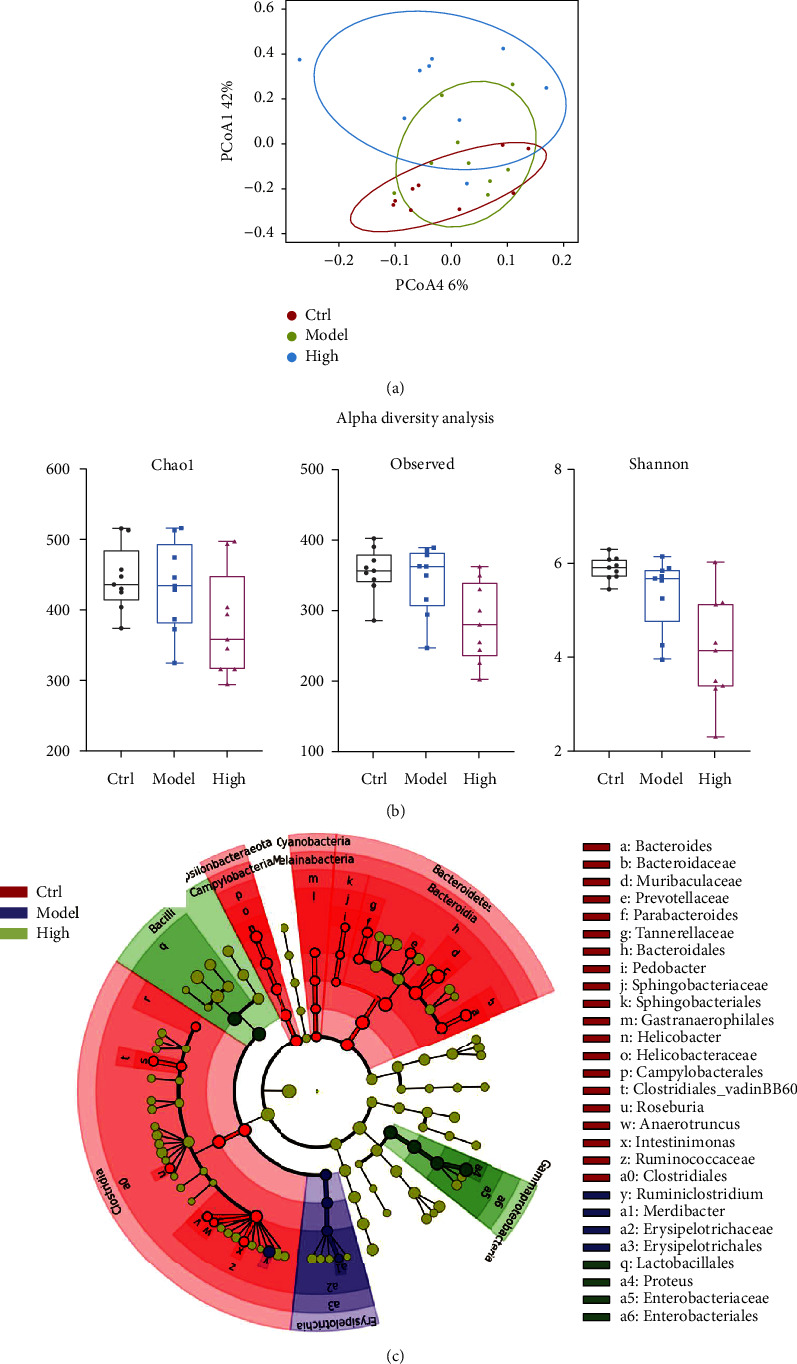
Treatment with high dosage of FFSLD could change the GM composition. (a) PCoA results of the 16S sequencing data. (b) Alpha diversity analysis for the 16S sequencing data. (c) LEfSe analysis for the 16S sequencing data. Ctrl for the control group group; model for the model group; high for the high dosage of FFSLD.

Previous results showed that the diabetic rats treated with high dosage of FFSLD displayed a better effect when compared to the low and middle dosage of FFSLD. Here, in order to investigate the changes in GM composition of FFSLD, 16S high-throughput sequencing was used to analyze the fecal GM composition among the control, model, and high dosage of FFSLD groups. PCoA result showed the distinct clustering among the control, model, and high dosage of the FFSLD groups (Figure 4(a)). Moreover, alpha diversity analysis showed that treatment with high dosage of FFSLD could dramatically decrease the Chao 1, Observe, and Shannon indexes when compared with the control and model groups (Figure 4(b)). In addition, LEfSe analysis result also displayed the significant difference of the GM composition among the mentioned groups. For instance, the model group showed the high relative abundance of the taxa of *Ruminiclostridium*, *Merdibacter*, and *Erysipelotrichaceae*. However, *Lactobacillales*, *Proteus*, and *Enterobacteriaceae* were significantly increased by treated with high dosage of FFSLD (Figure 4(c)).

### 3.4. GM Changed by High Dosage of FFSLD Correlated with the Diabetic-Related Serum Indicators and the Intestinal Metabolic Pathways in the T2DM Rats

**Figure 5:**
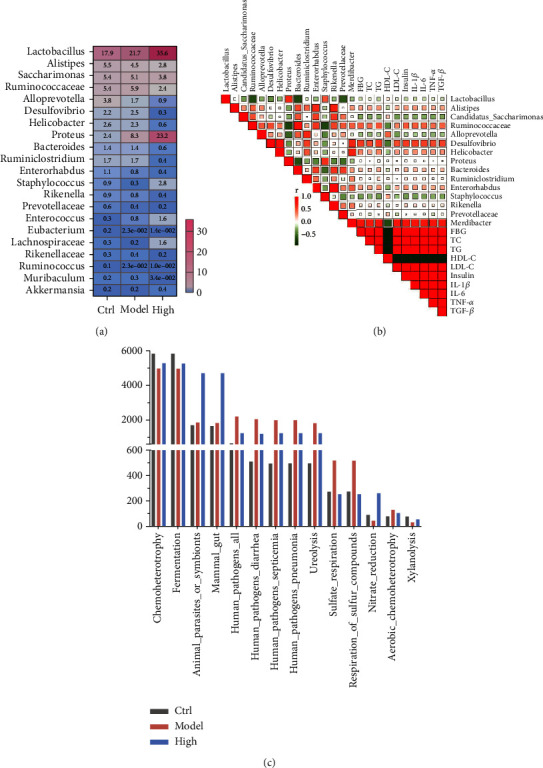
GM changed by a high dosage of FFSLD correlated with the diabetic-related serum indicators and the intestinal metabolic pathways. (a) Heatmap analysis for the genus among the control, model and high dosage of FFSLD groups. (b) Pearson's correlation analysis between GM and diabetic-related serum indicators. (c) FAPROTAX analysis for the intestinal metabolic pathways.

To further deconstruct the GM composition changed by high dosage of FFSLD, the taxa of key genera were selected for the analysis. Results showed that FFSLD significantly increased the relative abundance of certain beneficial bacteria (*Lactobacillus* and *Akkermasia*), and *Proteus* which was an important commensal GM in the host gastrointestinal tract. Meanwhile, the relative abundance of potential pathogens (*Alistipes*, *Desulfovibrio*, and *Helicobacter*) which were closely linked to gut inflammation has been found to decrease in the rats with FFSLD intervention (Figure 5(a)).

Moreover, Pearson's correlation analysis was used to comprehensively analyze the relationship among the diabetic biochemical indexes, proinflammatory cytokines, and the genera of gut microbiota. As shown in Figure 5(b), the five biochemical indexes (FBG, TC, TG, LDL-C, and insulin) and four proinflammatory cytokines (IL-1*β*, IL-6, TNF-*α*, and TGF-*β*) were negatively related to the genera of *Lactobacillus* and *Staphylococcus*, while positive related to *Alistipes*, *Desulfovibrio*, *Helicobacter*, and *Ruminococcaceae*. All these results suggested that the changed GM composition strongly correlated with the diabetic-related serum indicators (FBG, TC, TG, and LDL-C) and the proinflammatory cytokines, including IL-1*β*, IL-6, TNF-*β*, and TGF-*β*.

To further evaluate the interaction among the treatment, GM composition and intestinal metabolic pathways, FAPROTAX database was used to predict the abundance of pathways after being treated with FFSLD. The results showed that the high dosage of FFSLD treatment could increase the abundance of metabolic pathways including chemoheterotrophy, fermentation, nitrate reduction, and xylanolysis, while downregulating the pathways such as pathogens associated with diarrhea, septicemia, and pneumonia, as well as ureolysis, sulfate, and sulfur respiration (Figure 5(c)).

## 4. Discussion

In this study, Sprague–Dawley rats treated with STZ injection and HFD feeding were used to establish the T2DM model. Compared with the model group, rats treated with FFSLD showed a significant dose-dependent recovery in the biochemical indexes such as FBG, TC, TG, HDL-C, LDL-C, and insulin, which suggested the antidiabetic effect of FFSLD in the T2DM rats. Furthermore, the proinflammatory cytokines such as IL-1*β*, IL-6, TNF-*α*, and TGF-*β* were significantly decreased both in the serum and intestinal mucosa of FFSLD treated rats. In addition, GM composition of the diabetic rats changed by FFSLD was related to the diabetes-associated indicators and proinflammatory cytokines in serum and mucosa.

Diabetes is a metabolic disease characterized by disordered glucose metabolism and seriously threatens the quality and longevity of human life [19]. Patients with T2DM always show abnormal glucose and lipid metabolism in the clinic, including the high level of FBG, insulin, TC, TG, LDL-C, and the low level of HDL-C [24]. Metformin, the first-line treatment for diabetes, has been widely used for nearly sixty years [25]. It shows a good hypoglycemic effect and always be used as the positive control in many experimental studies, while it has adverse effects such as gastrointestinal tract (nausea and diarrhea) or vitamin B12 malabsorption after administration for a long time [26]. FFSLD consists of *Psidium guajava* L. (Fanshiliuye), *Ficus simplicissima* Lour. (Wuzhimaotao), *Euonymus alatus* (Thunb.) Sied. (Guijianyu), *Morus alba* L. (Sangye), *Codonopsis pilosula* N. (Dangshen), *Coptis chinensis* F. (Huanglian), *Atractylodes lancea* DC. (Cangzhu), *Zingiber officinale* R. (Ganjiang), *Crataegus pinnatifida* B. (Shanzha), and *Monascus purpureus* W. (Hongqu). A previous study showed that treatment with the combination of FFSLD and metformin could significantly decrease the FBG, HbA1c, TC, and TG in T2DM patients when compared with the patients treated with single metformin, which suggested the synergistic effect of FFSLD on T2DM clinical therapeutics [27]. In this study, FFSLD dose dependently reduced clinical indicators of T2DM. The antidiabetic effect of high-dose FFSLD has no significant difference when compared with metformin. In diabetic patients, the levels of inflammatory cytokines were found to increase in the blood, and these alternations completely modify the behavior of leukocytes. Long-term inflammatory stimulation contributes to maintaining insulin resistance and inhibiting insulin secretion, which is closely related to the diabetic complication [28]. Therefore, inhibition of inflammatory reaction as early as possible is the key therapeutic strategy for T2DM. Previous clinical research suggested that several small molecules specifically arrest proinflammatory pathways and effectively improved insulin sensitivity and lower blood glucose [29]. In our study, serum IL-1*β*, IL-6, TNF-*α*, and TGF-*β* were increased in the rats of the model group, and these factors were reduced by FFSLD dose dependently. Furthermore, the effects of FFSLD also displayed a similar tendency on inhibiting the expression of proinflammatory cytokines in the intestinal mucosa. Collectively, these results suggested FFSLD effectively alleviated inflammatory responses induced by diabetes.

Many factors such as hyperglycemia, oxidative stress, and lipotoxicity induce the inflammatory response and exacerbate the local inflammation [30]. GM, which consisted of 500–1000 different species, is recognized as a complex “organ” maintaining host health [31]. However, more and more studies have proved that gut dysbiosis plays an important role in T2DM and inflammatory reactions [32]. Generally, GM is divided into commensal bacteria, beneficial bacteria, and conditionally pathogenic bacteria (also mentioned as pathobiont), which participate in regulating the intestinal barrier, intestinal peristalsis, synthesis of vitamins, and reabsorption of substances [33]. Traditional Chinese medicine contains complex components and was administered through the gastrointestinal tract. Therefore, we speculate that the antidiabetic effect of FFSLD is closely related to reinstating the imbalance of GM composition in T2DM. Therefore, we speculated that FFSLD alleviates diabetic symptoms in T2DM rats through regulating and reinstating the imbalance of GM composition. In this study, GM composition was significantly changed in the FFSLD treated rats. Although, Chao 1, Observe, and Shannon indexes were dramatically decreased, the relative abundance of *Lactobacillus* and *Akkermasia* which was considered as the beneficial bacteria were increased with FFSLD treatment. Meanwhile, potential pathogens such as *Alistipes*, *Desulfovibrio*, and *Helicobacter* were inhibited by FFSLD. *Lactobacillus,* a well-known probiotic, has been found alleviating inflammatory response and oxidative stress both in obese mice [34] and aging mice [35]. Although the major probiotic sold on the market are mainly focused on the microorganisms from *Lactobacillus*, more and more studies have proved the investigation and application prospect of *Akkermasia muciniphila* [36]. The administration of *A. muciniphila* effectively reduces the weight of obese mice and restore the gut barrier function which decreases plasma LPS levels and inflammation [37]. The correlation analysis in this study showed the negative correlation of *Lactobacillus* and *Staphylococcus* with some of the physiological factors such as FBG, TC, TG, LDL-C, and insulin and proinflammatory cytokines (IL-1*β*, IL-6, TNF-*α*, and TGF-*β*), while *Alistipes*, *Desulfovibrio*, and *Helicobacte* showed positive correlation with these serum parameters. All these suggested FFSLD alleviated symptoms of T2DM and reduced inflammation through reshaping the GM.

## 5. Conclusion

In this study, FFSLD was found effectively alleviate the diabetic biochemical indexes via reducing the serum concentration of FBG, TC, TG, LDL-C, and insulin, and increasing the concentration of HDL-C in STZ injection combing HFD feeding T2DM rat model. Furthermore, the proinflammatory cytokines were reduced in FFSLD treated rats in a dose-dependent manner. Meanwhile, FFSLD significantly changed the GM of T2DM rats through regulating the relative abundance of *Lactobacillus*, *Akkermasia, Alistipes*, *Desulfovibrio*, and *Helicobacter*. Further study showed that these changed bacteria are closely related to the proinflammatory cytokines. All these results clarify the underlying mechanisms of FFSLD on the antidiabetic effect and provide a theoretical basis for its further clinical application.

## Data Availability

The raw data supporting the conclusions of this manuscript will be made available by the authors, without undue reservation, to any qualified researcher.

## Supplementary Materials

Supplementary Materials The supplementary materials included the method and result of Fufang Fanshiliu decoction (FFSLD) quality control. Figure S1: HPLC chromatograms of FFSLD sample solution.
