# Supplementary figures and images for: Fufang Fanshiliu Decoction Revealed the Antidiabetic Effect through Modulating Inflammatory Response and Gut Microbiota Composition
Source: Evid Based Complement Alternat Med. 2022 Oct 10;2022:3255401. doi: 10.1155/2022/3255401 (PMC9576391; doi:10.1155/2022/3255401)

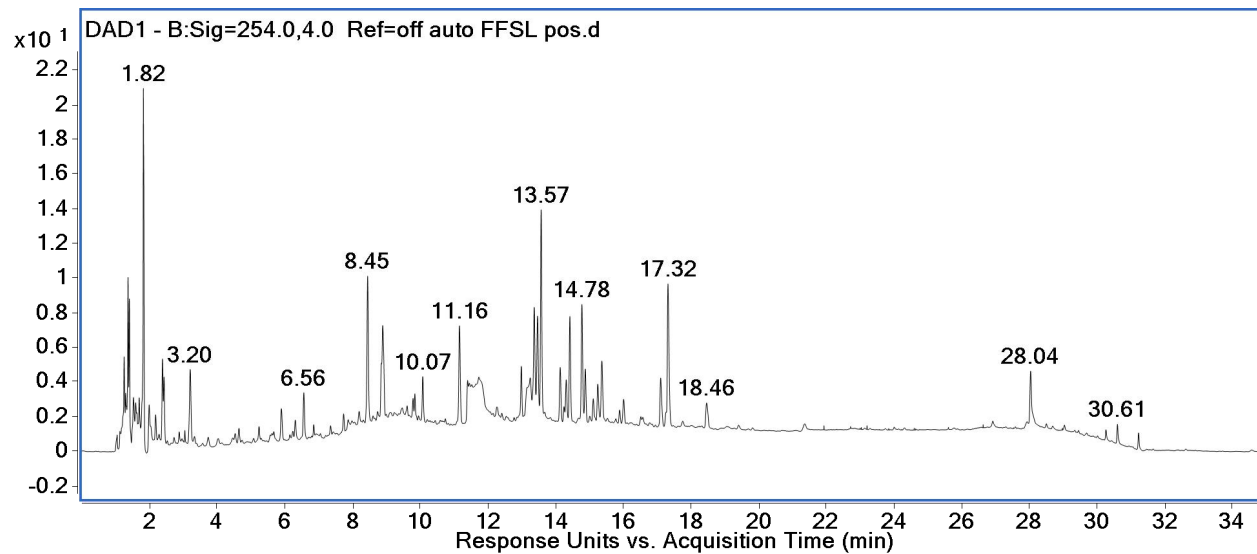

Supplement: Supplementary Materials — The supplementary materials included the method and result of Fufang Fanshiliu decoction (FFSLD) quality control. Figure S1: HPLC chromatograms of FFSLD sample solution. [file 3255401.f1.zip › 3255401.f1/S1 (1).pdf]
