# Supplementary material for: Fufang Fanshiliu Decoction Revealed the Antidiabetic Effect through Modulating Inflammatory Response and Gut Microbiota Composition
Source: Evid Based Complement Alternat Med. 2022 Oct 10;2022:3255401. doi: 10.1155/2022/3255401 (PMC9576391; doi:10.1155/2022/3255401)
Supplement: Supplementary Materials — The supplementary materials included the method and result of Fufang Fanshiliu decoction (FFSLD) quality control. Figure S1: HPLC chromatograms of FFSLD sample solution. [file 3255401.f1.zip › 3255401.f1/supplementarial file-upload (1).doc]

**Fufang Fanshiliu decoction revealed the anti-diabetic effect through modulating inflammatory response and gut microbiota composition-Supplementarial file**

Leyu Li ^a1^, Guoxin Huang ^b1^, Tingbo Chen^c^, Hui Lin^a^, Ruiyan Xu^a^, Jinyan Cheng^a^, Ying Hu^a^, Weibo Dai^a*^, Gengting Dong^a*^

a Pharmacology Laboratory, Zhongshan Hospital of Traditional Chinese Medicine, Zhongshan, Guangdong, China

b Clinical Research Center, Shantou Central Hospital, Shantou, Guangdong, China

c College of health industry， Zhongshan Torch Polytechnic, Zhongshan, Guangdong, China

1 These authors contributed equally to this work.

* Corresponding authors:

Gengting Dong, Zhongshan, Guangdong, China, Email address: donggengting@163.com;

Weibo Dai, Zhongshan, Guangdong, China, Email address: [daiweibo007@163.com](mailto:daiweibo007@163.com)

**1. The UHPLC-DAD analysis of FFSLD**

**1.1 Method**

FFSLD powder was dissolved in methanol/water (1:4, v/v) solution at 55.2 mg/ml. The mixture was sonicated for 40 min, and then centrifuged at 13000 rpm for 10 min. The supernatant was collected and filtered to be the final FFSLD sample solution. UHPLC-DAD assay of FFSLD was performed on a Agilent UHPLC system (1290, Agilent, United States) equipped with a binary solvent delivery system, an auto-sampler and column manager. UHPLC Chromatographic separation was performed on a C18 column (3.0*150mm, 1.8um, Zorbax). The mobile phase consisted of 0.1% (v/v) formic acid buffer (solvent A) and acetonitrile (solvent B). Elution procedure was 0~2 min, 5% B, 2~20min, 5%~35% B, 20~24min, 35%~40% B, 24~25min, 40%~50% B, 25~30min, 50%~95% B, 30~35min, 95% B. The flow rate was 0.5 ml/min with a column temperature of 30 ℃. The injection volume was 0.5μl in full loop mode. UV absorbance data were collected at 245 nm.

**1.2 Result**

Precision FFSLD sample solution was analyzed according to the method of chromatographic condition described in 1.1. Many chromatographic peaks recorded (S1) by UHPLC-DAD could be used for the quality control of FFSLD. And the future research of QTOF-MS was also carried out for compounds identification. The results showed that 457 compounds were identified from FFSLD which was reported in previously described [1].

S1 HPLC chromatograms of FFSLD sample solution.

**Reference**

[1]	W. Dai, C. Chen, G. Dong, et al., “Alleviation of Fufang Fanshiliu decoction on type II diabetes mellitus by reducing insulin resistance: A comprehensive network prediction and experimental validation,” *J Ethnopharmacol*, vol. 294, p. 115338, Aug. 2022.
